# Functionalization of α-In_2_Se_3_ Monolayer via Adsorption of Small Molecule for Gas Sensing

**DOI:** 10.3389/fchem.2018.00430

**Published:** 2018-09-26

**Authors:** Zhi Xie, Fugui Yang, Xuee Xu, Rui Lin, Limin Chen

**Affiliations:** ^1^College of Mechanical and Electronic Engineering, Fujian Agriculture and Forestry University, Fuzhou, China; ^2^School of Electronic Information Science, Fujian Jiangxia University, Fuzhou, China

**Keywords:** 2D materials, first-principles calculation, In_2_Se_3_, charge transfer, gas sensor

## Abstract

Based on first-principles calculations, the adsorption of NO and NO_2_ gas molecules on the α-In_2_Se_3_ monolayer have been studied. The adsorption configuration, adsorption energy, electronic structure and charge transfer properties are investigated. It is found that the charge transfer processes of NO and NO_2_ adsorbed on the surface of α-In_2_Se_3_ monolayer exhibit electron donor and acceptor characteristics, respectively. After the adsorption of the molecules, the α-In_2_Se_3_ monolayers have new states near the Fermi level induced by NO and NO_2_, which can trigger some new effects on the conducting and optical properties of the materials, with potential benefits to gas selectivity. The present work provides new valuable results and theoretical foundation for potential applications of the In_2_Se_3_-based gas sensor.

## Introduction

In recent years, Layered two-dimensional (2D) materials have received tremendous research attention due to their unique physical and chemical properties (Miró et al., 2014; Bhimanapati et al., 2015; Xie et al., 2018). Because of their ultrahigh flexibility, strength and thickness-dependent electronic properties (Wang et al., 2012; Novoselov et al., 2016), the nanodevices based on 2D materials and tuning the properties of their heterostructures via defects engineering (Cervenka et al., 2009; Wang et al., 2011; Park et al., 2014; Sun et al., 2015) hold great promise for potential applications in nanoscale electronics, optoelectronics and spintronics (Wang et al., 2008, 2016; Geim and Grigorieva, 2013; Lan, 2018). Additionally, the high surface/volume ratio, weak electronic screening and ultrathin thickness of 2D materials induce that their structural stability and electronic properties are very sensitive to environmental molecules, and the relevant effects make them efficient for gas molecules sensing, catalysis, and energy storage technologies (Lightcap and Kamat, 2013; Yang et al., 2016; Zhang et al., 2018). Graphene has exhibited good performance in the field of gas sensor (Kemp et al., 2013). Previous reports have also shown that MoS_2_-based nanosensors possess excellent sensing ability with high response value, and their molecule adsorption properties can be modulated by applying light, strain, and external electric field (Late et al., 2013; Ma et al., 2016). Recently, InSe monolayer has been found having tunable electronic properties via the molecule adsorption and promising for gas sensing application (Ma et al., 2017). All these studies clearly reveal that external factors can modulate the properties of 2D materials effectively and extend their application fields.

Indium selenide (In_2_Se_3_) is an interesting III-VI group layered chalcogenide compound with multiple phases and excellent properties (Shi et al., 2013), and have attracted extensive research interest for the applications in phase change memory (Yu et al., 2007), lithium batteries (Feng et al., 2016), optoelectronic and photovoltaic devices (Zhai et al., 2010; Jacobs-Gedrim et al., 2014). Among all the phases, 2D materials based on α-In_2_Se_3_ exhibit obvious thickness-dependent shift of band gap and promising prospects for tunable wavelength photodetection (Quereda et al., 2016). It is also reported that the strain sensor fabricated from 2D α-In_2_Se_3_ films possesses good stability, excellent sensitivity, and high spatial resolution in strain distribution, showing attractive properties for e-skin applications in wearable electronics (Feng et al., 2016). However, to best of our knowledge, the investigations on the adsorption of small gas molecules on atomically thin 2D In_2_Se_3_ materials and the related modification of their properties are still lacking so far. It is well-known that NO_2_ and NO are common air pollutants and harmful to human health. The detection and control of them are very important for the environmental protection. Hence, in this work, we have made first-principles studies on the α-In_2_Se_3_ monolayers adsorbed by NO and NO_2_, respectively. The adsorption configuration, structural stability, electronic structure and charge transfer properties have been investigated and discussed in detail.

## Computational methods

All calculations are carried out using the Vienna *ab initio* simulation package (VASP)(Kresse and Furthmüller, 1996), with the core electrons described by the projected augmented wave (PAW) method. For the exchange-correlation term, the generalized gradient approximation (GGA) with Perdew-Burke-Ernzerhof (PBE) scheme is employed. The cutoff energy for plane-wave basis is set as 450 eV. For simulating the adsorption of the molecules, a 4 × 4 × 1 supercell of the α-In_2_Se_3_ monolayer is built with one NO or NO_2_ molecule adsorbed on its surface, and a vacuum space of more than 15 Å is set up to prevent the interactions between the repeated monolayers. The Monkhorst-Pack of 2 × 2 × 1 (4 × 4 × 2) *k*-point grid is adopted for the Brillion zone sampling in geometry optimization (total energy calculation). The convergence criterion of energy is taken as 10^−5^ eV. Structure relaxation is performed until the force on each atom is smaller than 0.02 eV/Å. To estimate the adsorption stability of gas molecules on the surface of α-In_2_Se_3_ monolayer, the adsorption energy (E_ad_) is calculated by the formula: E_ad_ = E_M_ + E_G_ – E_M+G_, where E_M_, E_G_, and E_M+G_ denote the total energy of the α-In_2_Se_3_ monolayer, the free gas molecule, and the α-In_2_Se_3_ monolayer adsorbed by gas molecules, respectively. According to this definition, a positive value of E_ad_ represents the adsorption is energetically favorable.

## Results and discussion

**Figure 1:**
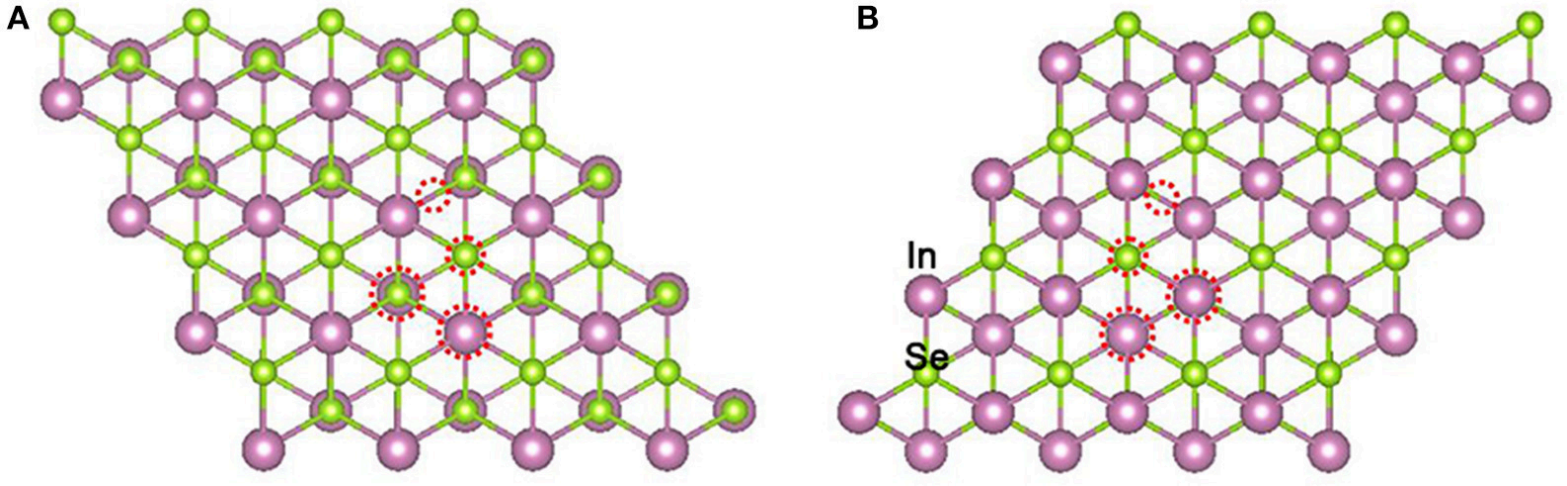
Top views of typical adsorbing sites on the Se atom planes of one side **(A)** and the other side **(B)** of the α-In_2_Se_3_ monolayer. The adsorbing sites are denoted by red dotted circles.

Firstly, the geometry optimizations of free gas molecules were performed. The obtained bond lengths of NO and NO_2_ are 1.17 and 1.21 Å, respectively, and the O-N-O bond angle of NO_2_ is 133.39°. The band gap of the clean α-In_2_Se_3_ monolayer has been calculated to be 0.77 eV (see **Figure 3A**). All these results are in line with the data of previous reports (Debbichi et al., 2015; Ma et al., 2017). In order to find the most stable adsorption configuration, four typical adsorbing sites on the Se atom plane of one side have been considered including the top of Se atom, the center of a Se-In bridge and two centers of the hexagonal void (see Figure 1A). Because of the different coordination structures of the Se atom plane on the other side, four similar adsorbing sites were also investigated on the other side (see Figure 1B).

**Figure 2:**
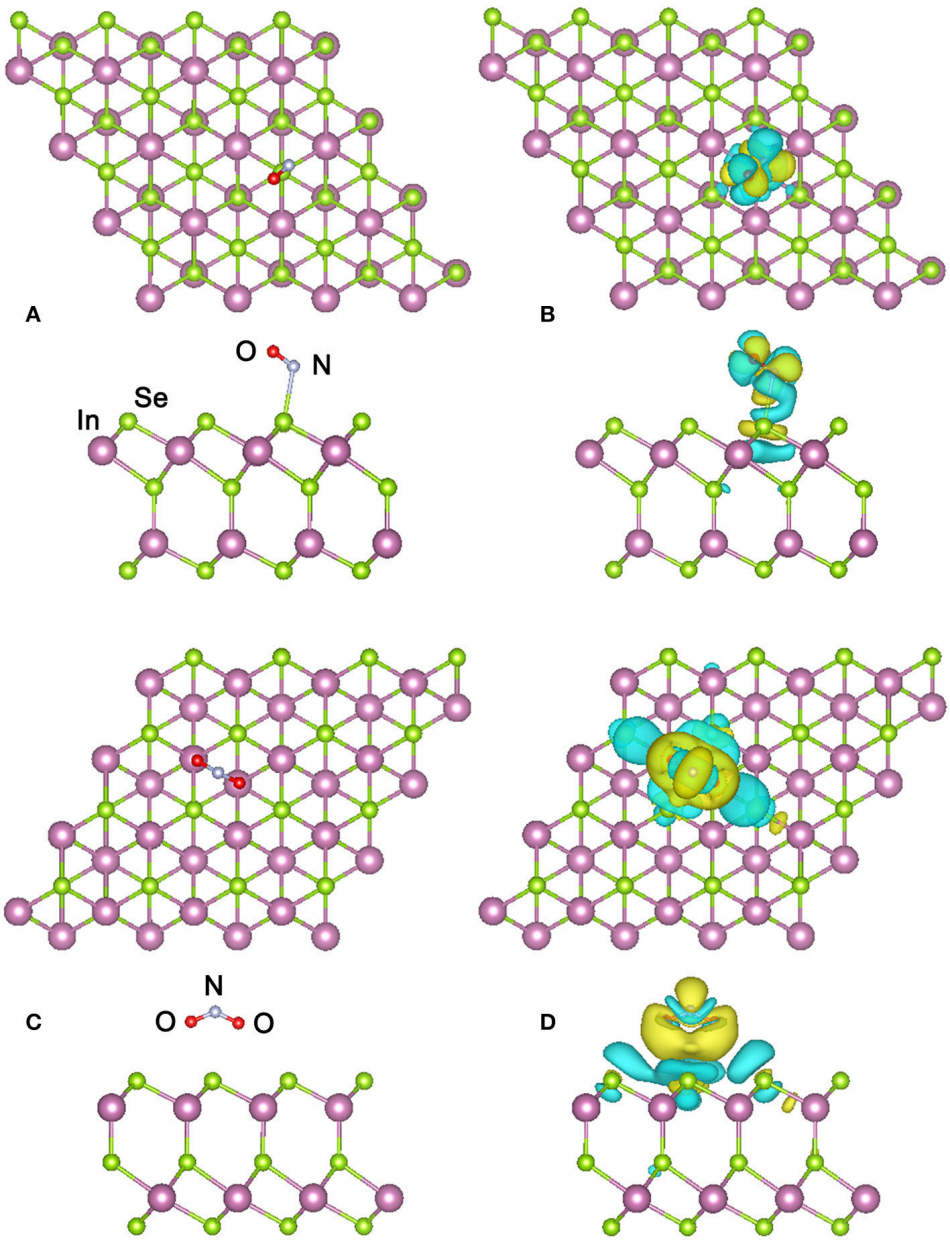
**(A)** Top and side views of the most stable configuration of the α-In_2_Se_3_ monolayer adsorbed by NO and **(B)** its charge density difference. **(C)** Top and side views of the most stable configuration of the α-In_2_Se_3_ monolayer adsorbed by NO_2_ and **(D)** its charge density difference. The cyan and yellow isosurfaces denote the electron depletion and accumulation, respectively. The isosurface value is set as 0.0001 e/bohr^3^.

For the adsorption of NO molecule, besides the eight adsorbing sites mentioned above, we also considered two different orientations of the molecule with the N-O bond perpendicular or parallel to the surface of α-In_2_Se_3_ monolayer. Therefore, 16 configurations have been examined. Figure 2A presents the top and side views of the most stable configuration obtained, where the O atom of NO molecule points away from the α-In_2_Se_3_ surface and the N atom of NO molecule points toward the surface with the smallest distance between the adsorbed NO and the surface atom is 2.65 Å. The N-O bond is a little shortened to 1.16 Å compared with that (1.17 Å) of free NO molecule. The adsorption energy was calculated to be 208 meV, which is comparable to those of NO adsorptions on the monolayers of InSe, GaSe, and MoS_2_ (Yue et al., 2013; Ma et al., 2017; Zhou et al., 2017). This low adsorption energy indicates the NO adsorption capability of α-In_2_Se_3_ monolayer is not very strong, which is applicable for the gas detection since the adsorption-desorption of NO molecule on α-In_2_Se_3_ monolayer can be easily achieved.

**Figure 3:**
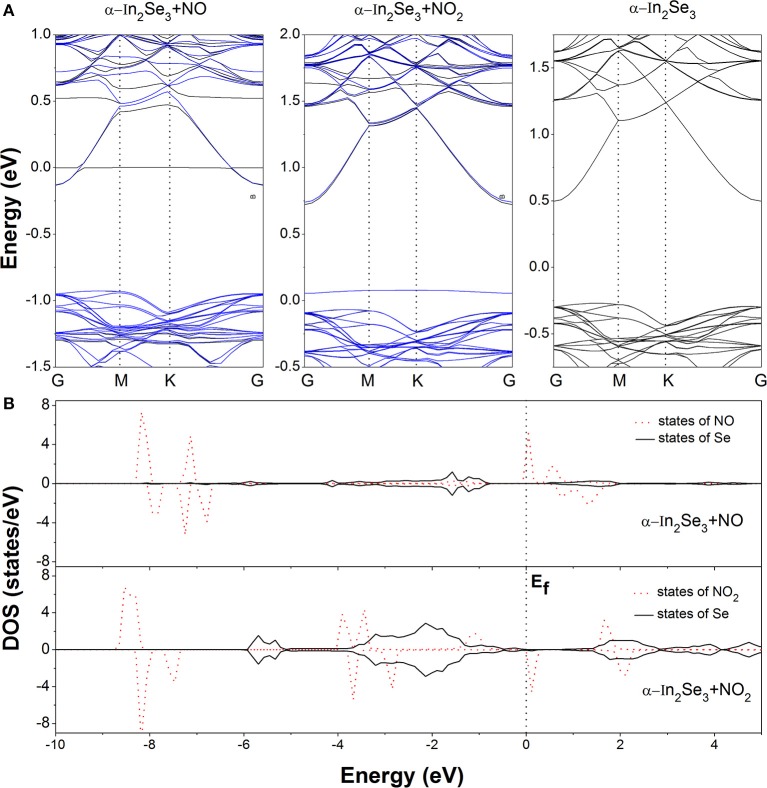
**(A)** The band structures of the clean α-In_2_Se_3_ monolayer and the most stable configurations of the α-In_2_Se_3_ monolayer adsorbed by NO and NO_2_. For the two adsorption cases, the black and blue curves represent the spin up and spin down bands, respectively. **(B)** The local density of states (DOS) of the adsorbed molecules and the Se atoms near them. The Fermi level is set as 0 eV.

For further investigating the interactions and electron transfers between the adsorbed NO molecule and the α-In_2_Se_3_ monolayer, the charge density difference (CDD) is calculated from the formula: Δρ = ρ_*M* + *G*_ − ρ_*M*_−ρ_*G*_, where ρ_*M*+*G*_, ρ_*M*_ and ρ_*G*_ represent the total charge densities of the α-In_2_Se_3_ monolayer adsorbed by gas molecules, the α-In_2_Se_3_ monolayer, and the isolated gas molecule, respectively. The ρ_*M*_ and ρ_*G*_ are obtained with each atom at the same position as the adsorption configuration. In the NO_2_ adsorption case, the similar calculation has also been performed. As shown in Figure 2B, it can be seen that the adsorption make the redistribution of charges around the NO molecule. In the space between the adsorbed NO and the α-In_2_Se_3_ surface, the depletion of electrons is dominant. Based on the Bader charge analysis, the charge transfer has been quantitatively calculated. It is demonstrated that the NO molecule provides 0.054 *e* electrons to the α-In_2_Se_3_ surface and acts as an electron donor. This behavior is different from the situation of the NO adsorption on InSe monolayer, where the NO molecule acts as an electron acceptor with the amount (0.018 *e*) of transferred charges (Ma et al., 2017) smaller than that (0.054 *e*) between NO and α-In_2_Se_3_ monolayer. The band structure of the most stable configuration is depicted in Figure 3A, it is shown that after the NO adsorption the Fermi level (E_f_) of the system moves upwards to the bottom of the conduction bands compared with that of the clean α-In_2_Se_3_ monolayer (see Figure 3A), demonstrating an n-type conducting property of the materials with NO adsorption, which is similar to the situation of property modification in the NO-adsorbed MoS_2_ monolayer (Shokri and Salami, 2016). This property changes can be useful to the detection of NO molecule. In addition, some new states are found to be located at the E_f_. To better understand the adsorption effect of NO molecule on the α-In_2_Se_3_ monolayer, the local density of states (DOS) of the adsorbed NO and its nearest Se atom are illustrated in Figure 3B. It is clearly shown the new states at the E_f_ are from the adsorbed NO, and there is little hybridization between the states of NO molecule and the states of the surface Se atom near it, which is similar to the NO adsorption behavior on InSe monolayer, further confirming that the interaction between the adsorbed NO molecule and the α-In_2_Se_3_ monolayer is not strong.

In the NO_2_ adsorption case, two orientations of NO_2_ molecule have been considered. One is the two O atoms point toward the α-In_2_Se_3_ surface, and the other is that they point away from the α-In_2_Se_3_ surface. The obtained most stable adsorption configuration is displayed in Figure 2C, in which the NO_2_ is adsorbed on the Se atom plane of the other side different from that of the NO adsorption case and the two O atoms of NO_2_ molecule point toward the α-In_2_Se_3_ surface. The smallest distance between the NO_2_ molecule and the α-In_2_Se_3_ surface is 3.57 Å, and the N-O bond is a little elongated to 1.22 Å with the O-N-O angle reduced to 131.89°. The adsorption energy was calculated to be 59 meV, suggesting the adsorption of NO_2_ on α-In_2_Se_3_ monolayer is weak, which is comparable to that of the NO_2_ adsorption on graphene (Leenaerts et al., 2008).

The CDD of the most stable configuration for NO_2_ adsorption is displayed in Figure 2D. It is shown that the charge redistribution of NO_2_ molecule is apparent. The electrons accumulate in the vicinity of the adsorbed NO_2_ with a little electron depletion in its core region. The depletion of electrons mainly occurs for the Se atoms around the NO_2_ molecule. From the Bader analysis, it is indicated that the NO_2_ molecule is an electron acceptor and obtains 0.081 *e* electrons from the α-In_2_Se_3_ monolayer, which is similar to the situation of NO_2_ adsorption on InSe monolayer, and their amounts of transferred charges are comparable (Ma et al., 2017). The band structure of the discussed configuration is shown in Figure 3A. It can be seen that there is a new impurity band lying just above the top of the valence bands and the E_f_, which can modify the optical and conducting properties of the materials, benefiting the detection of NO_2_ molecule. As shown in Figure 3B, the local DOS distributions demonstrate that the impurity states just above the E_f_ are from the adsorbed NO_2_ molecule, and there are some overlaps of states between the NO_2_ molecule and the Se atoms near it.

## Conclusion

To explore the gas sensing applications of 2D materials based on In_2_Se_3_, the effects of the adsorbed NO and NO_2_ molecules on α-In_2_Se_3_ monolayer have been studied using first-principles calculations. When the NO and NO_2_ are adsorbed on the surface of the α-In_2_Se_3_ monolayer, the calculated adsorption energies of positive value indicate their adsorption processes are exothermic and energetically favorable. Their low adsorption energies demonstrate the α-In_2_Se_3_ monolayer is applicable for the gas molecules detection. In the most stable configurations, the gas molecules are adsorbed on different Se atom planes for NO and NO_2_, respectively, and the smallest distance (3.57 Å) between the adsorbed NO_2_ and the α-In_2_Se_3_ monolayer is larger than that (2.65 Å) of NO adsorption case. NO provides 0.054 *e* electrons to the α-In_2_Se_3_ monolayer as the donor gas molecule, while NO_2_ acts as the acceptor gas molecule and gains 0.081 *e* electrons from the α-In_2_Se_3_ monolayer. Both of the adsorbed molecules induce new electronic states near the Fermi level compared with the electronic structure of clean α-In_2_Se_3_ monolayer. These changes of electronic properties can modify the conducting and optical properties of the materials and benefit gas sensing. The theoretical findings of this work suggest the 2D α-In_2_Se_3_ materials hold great promise for the application of gas sensor.

## Author contributions

ZX and FY performed the calculations and analyzed the data with the help of XX, RL, and LC. ZX and FY wrote the manuscript with input from all authors. All authors read and approved the manuscript.

### Conflict of interest statement

The authors declare that the research was conducted in the absence of any commercial or financial relationships that could be construed as a potential conflict of interest.
